# Seed bacterial microbiota: transmission, community assembly, and prospects for engineering heritable functions in crops

**DOI:** 10.1016/j.crmicr.2026.100647

**Published:** 2026-07-18

**Authors:** Hamed Azarbad, Mehrdad Alizadeh

**Affiliations:** a Department of Biology, Evolutionary Ecology of Plants, Marburg University, Karl-von-Frisch-Strasse, 835043, Marburg, Germany; b Microbial Biogeochemistry, Research Area Landscape Functioning, Leibniz Centre for Agricultural Landscape Research e.V. (ZALF), 15374, Müncheberg, Germany; c Department of Plant Pathology, Faculty of Agriculture, Tarbiat Modares University, Tehran, Iran

**Keywords:** Seed microbiota, Vertical transmission, Microbial inheritance, Plant–microbe interactions, Microbiome engineering

## Abstract

Seeds represent specialized, low-biomass microbial niches that facilitate the transmission of the plant-associated microbiota from one generation to another. Residing as both epiphytes and endophytes, seed-associated bacteria contribute to microbial community assembly during the seed-to-seedling transition. Recent integration of single-seed omics and synthetic bacterial communities has moved the field beyond descriptive surveys to identify the microbial drivers of plant germination success and early-life resilience. Despite this potential, the establishment and persistence of seed-borne bacteria are often constrained by competition with soil-derived microbiota. Seedling bacterial community assembly is further shaped by host genotype, domestication history, and maternal environmental conditions. In this review, we synthesize the mechanisms of bacterial transmission from flowering through seedling emergence and evaluate the functional capacity of the seed holobiont. We define the ecological parameters for the successful recruitment of engineered seed-based consortia into the soil-plant continuum, providing an experimental roadmap to move seed-based technologies from the laboratory to the field. Ensuring the functional stability of these heritable microbial traits will be essential for stabilizing agricultural productivity under increasing climatic volatility.

## Highlights

• Seeds can transmit a subset of associated bacteria to the next plant generation.
• Seed-associated bacteria can influence germination, nutrient acquisition, and pathogen suppression.
• Seed-focused SynComs enable tests of bacterial persistence, seed-to-seedling transfer and effects on crop traits.

## Graphical abstract

**Figure:**
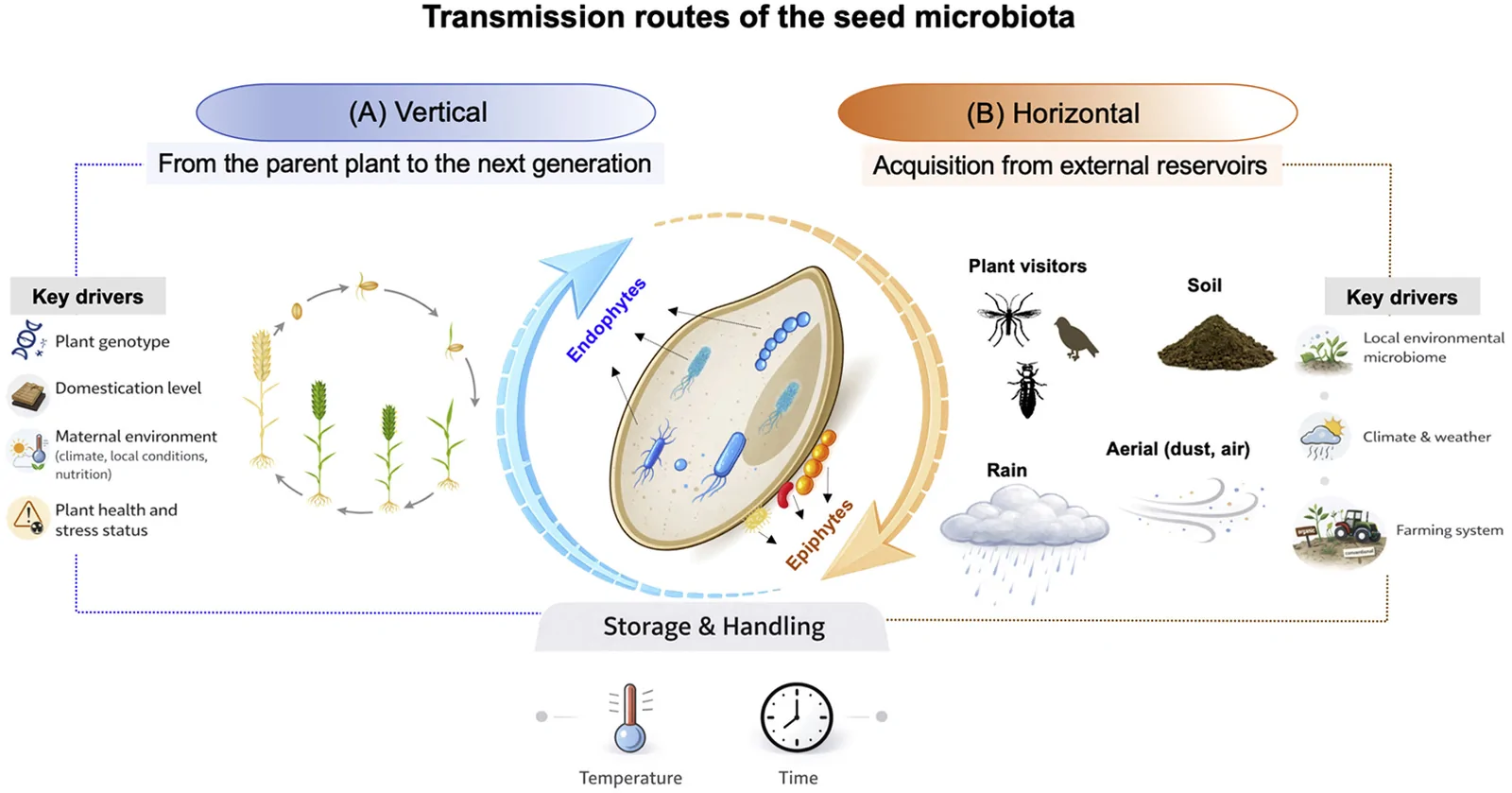

## 1 Introduction

Plants function as a holobiont, meaning that plant traits emerge from the plant plus the microorganisms that colonize compartments such as the rhizosphere, phyllosphere, and endosphere (Zilber-Rosenberg and Rosenberg, 2008; Mesny *et al.*, 2023; Poupin *et al.*, 2023). These microbes can influence nutrient acquisition, stress tolerance, and pathogen defence, and therefore contribute to plant performance across environments (Trivedi *et al.*, 2020; Xu *et al.*, 2021; Zai *et al.*, 2021; Azarbad and Junker, 2024; Compant *et al.*, 2024; Alizadeh *et al.*, 2026). Within the broader plant microbiome, seeds constitute a unique and still underexplored microbial habitat that connects one plant generation to the next (Berg and Raaijmakers, 2018; Abdelfattah *et al.*, 2022; Azarbad *et al.*, 2022, 2026; Tabassum *et al.*, 2024; Romão *et al.*, 2025). The seed microbiota, comprising bacteria, fungi, and archaea, can act as the earliest microbial inoculum for seedlings, thus influencing the assembly of subsequent plant microbiota (Hardoim *et al.*, 2012; Rodríguez *et al.*, 2018; Vujanovic *et al.*, 2019; Matilla, 2025; Xu *et al.*, 2026*b*). However, our mechanistic understanding of how seed-associated microbes are acquired, transmitted to the next plant generation, and function following seed exposure to soil remains limited.

Seed-associated bacteria colonize both the seed surface (epiphytes) and internal seed tissues (endophytes) (Hardoim *et al.*, 2015; Nelson, 2018; Azarbad *et al.*, 2022, 2026), where specific taxa undergo vertical transmission from the maternal host (Kim *et al.*, 2022*b*; Zhang *et al.*, 2022; Sulesky-Grieb *et al.*, 2024). Across diverse crop species, some bacterial taxa such as *Pantoea, Massilia, Pseudomonas,* and *Sphingomonas* are commonly detected, suggesting a broad host-range persistence in seed-borne communities (Zhang *et al.*, 2022; Wu *et al.*, 2024; Sanz-Puente *et al.*, 2025; Azarbad *et al.*, 2026). In terms of functional relevance for plants, some members of seed-associated bacteria have been shown to modulate germination dynamics and early seedling vigor through phytohormone signaling, nutrient mobilization, and pathogen antagonism (Santoyo *et al.*, 2016, 2025; Bergna *et al.*, 2018; Matsumoto *et al.*, 2021; Sanz-Puente *et al.*, 2025).

During germination, seed exudates create the spermosphere (zone surrounding a germinating seed enriched by seed-derived metabolites), which promotes microbe–microbe interactions and mediates the early stages of seedling colonization (Schiltz et al., 2015; Pitzschke, 2018). Although the seed microbiota can limit pathogen invasion during seed emergence, the extent of this protective effect depends on host genotype, pathogen virulence, and prevailing environmental conditions (Jack and Nelson, 2018; Wang *et al.*, 2023; War et al., 2023; Xun *et al.*, 2023). Climate change strengthens the need to understand seed microbiota because heat and drought can impair seed emergence when seedlings have limited capacity to compensate (Seleiman *et al.*, 2021; Sato *et al.*, 2024). Seed-associated microorganisms are present during emergence and respond to resource release during germination, and in some cases may also carry intergenerational signatures associated with more stable seedling establishment (Azarbad *et al.*, 2026). These findings provide a clear rationale for testing whether seed microbiota can be harnessed to improve early plant performance under increasing environmental variability.

Single-seed approaches, gnotobiotic systems, and synthetic communities (SynComs) enable the causal validation of seed-borne microbial functions and their effects on plant phenotypes (Chesneau *et al.*, 2022; Simonin *et al.*, 2023; Arnault *et al.*, 2024). Nevertheless, empirical evidence for the stable intergenerational transmission of seed-associated microbiota remains limited. Most studies document taxonomic overlaps across life cycles, rather than tracking the persistence of specific bacterial strains across generations (Mitter *et al.*, 2017; Sanz-Puente *et al.*, 2025). This distinction matters because seeds simultaneously transmit vertically transmitted microbial taxa and inherited host epigenetic states shaped by parental environments (Portal-Gonzalez *et al.*, 2025; Azarbad *et al.*, 2026; Lopes *et al.*, 2026). Characterizing these two distinct pathways is necessary to understand how microbial and plant memory independently or interactively shape plant performance across generations, in particular under climate change stressors.

This review synthesizes current advances in seed microbiota research with a primary focus on bacteria. First, we evaluate evidence for transmission and horizontal acquisition to epiphytic and endophytic compartments. Next, we examine how seed bacterial communities assemble from flowering through storage and germination, and we assess functional evidence linking seed bacteria to early growth, stress tolerance, and pathogen suppression. We further discuss how seed-associated bacterial isolates and seed-focused SynCom experiments can test establishment on seeds, transfer to seedlings, and effects on plant phenotypes. Throughout this review, we highlight key knowledge gaps, particularly regarding multigenerational tracking of seed-associated microbes, disentangling microbial and host epigenetic contributions, and evaluating the persistence of microbiome-mediated effects under field conditions and soil community coalescence.

## 2 Transmission and community assembly of seed-associated bacteria

**Fig. 1:**
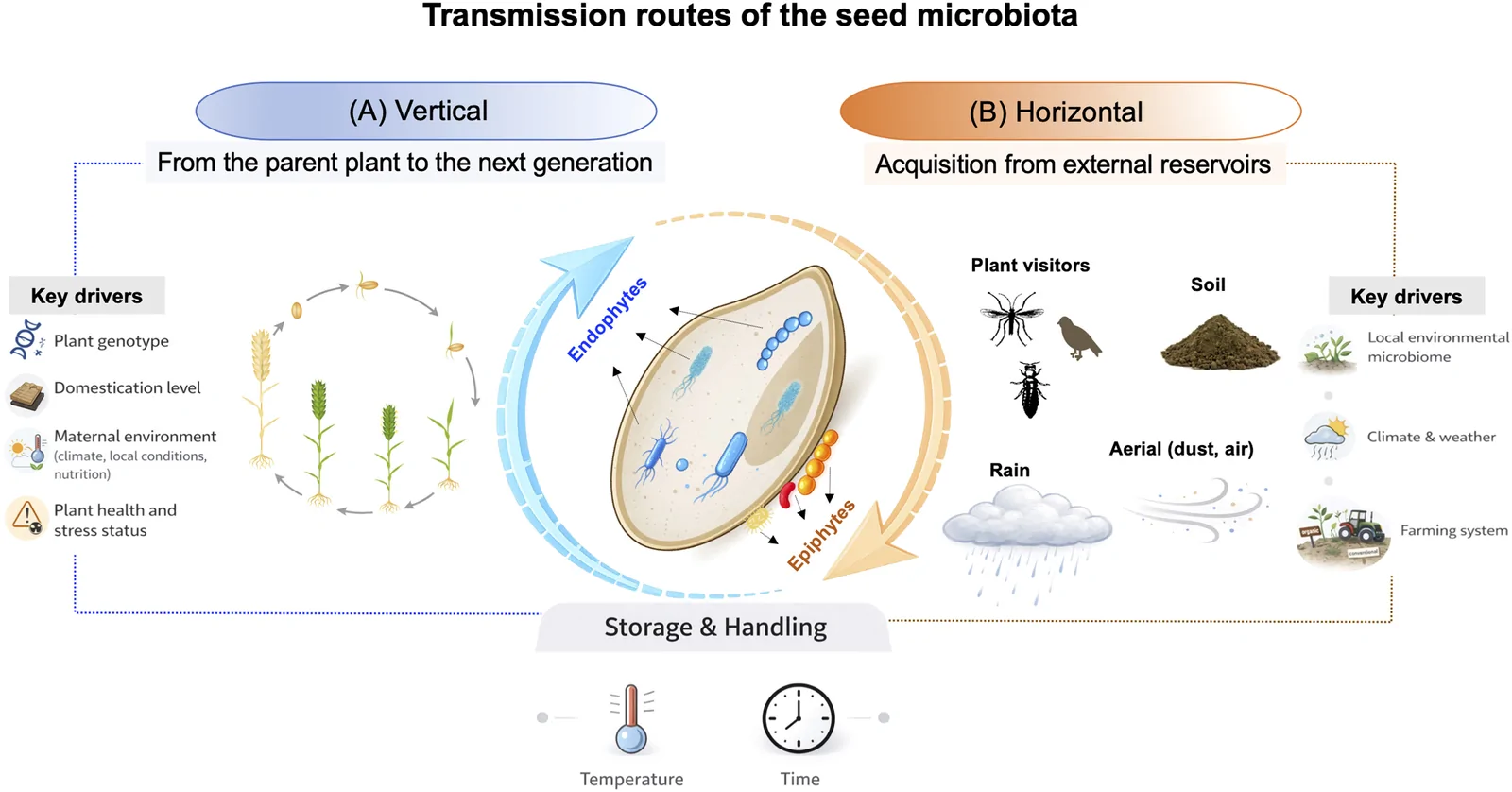
Transmission routes of seed-associated bacteria. Vertical transmission represents parent-to-offspring transfer of seed-associated microbes (A). Vertical transmission signals are shaped by host genotype, domestication history, and the maternal environment during flowering and seed maturation (including local climate), and can be modulated by plant health and stress status. Horizontal acquisition reflects microbial entry from external reservoirs (for example, soil, aerosols/dust, rainfall, and plant visitors such as insects) (B). In both routes, seed compartments (surface epiphytes versus internal endophytes) act as filters that can shift which taxa remain detectable and which members contribute during germination. The strength and composition of this bacterial input to seeds are expected to depend on the local environmental microbiome, climate conditions, and farming system. Across both routes, post-harvest storage and handling act as an additional filter that can restructure the seed microbiota and the subset of taxa available to contribute during germination, with time and temperature highlighted as major determinants.

Seed-associated bacterial communities can assemble through vertical transmission and horizontal acquisition (Fig. 1). Vertical transmission refers to the transfer of microorganisms from the parent plant to offspring seeds, potentially enabling persistence across plant generations, while horizontal acquisition is defined as if seeds acquire microbes from environmental reservoirs during the development stage or after dispersal (Edwards *et al.*, 2015; Shahzad *et al.*, 2018; War et al., 2023). In this part, we evaluate evidence for each route. Where possible, we distinguish seed epiphytes from seed endophytes because these fractions differ in exposure history and in their likelihood of persistence and transmission (Azarbad *et al.*, 2026).

### 2.1 Vertical transmission and multigenerational persistence

Across cereals, current evidence suggests that vertical transmission applies to only a restricted subset of seed-associated taxa rather than the entire seed microbial community (Kim *et al.*, 2022*a*; Sanz-Puente *et al.*, 2025). In rice, a large-scale field survey across two generations, six varieties, five plant microhabitats, and four locations identified a persistent core of ASVs, including ASVs assigned to the genera *Pantoea* and *Xanthomonas*, and reported high genomic similarity between parental and offspring seed isolates for these taxa, providing strong evidence for vertical transmission (Zhang *et al.*, 2022). Complementing this, a season-to-season study in rice identified 29 bacterial and 34 fungal taxa transmitted from seed to seed and reported that parental seeds and stem endospheres are major sources for progeny seed communities (Kim *et al.*, 2022*a*). Beyond cereals, similar evidence has also been reported in *Phaseolus vulgaris* L., a three-generation study demonstrated the vertical transmission of core seed-borne microbiota, identifying 22 endophytic bacterial taxa that persistently colonized the seed endosphere regardless of maternal drought or nutrient stress (Sulesky-Grieb *et al.*, 2024).

The seed compartment can act as a biological filter that regulates the composition of the inherited microbiota. We define microbial inheritance as the persistent maintenance of specific bacterial strains within the seed throughout the host life cycle (Fig. 1). In rice, seed-coat removal altered the bacterial and fungal communities detected in seeds and seedlings, indicating that the seed coat acts as a distinct microbial niche. The same study also tracked seed-associated taxa across the rice life cycle, including detection in leaf, stem, and root endospheres (Kim and Lee, 2021). In parallel, compartment-specific profiling of grain, outer grain, husk and outer husk revealed distinct bacterial and fungal communities and compartment-associated assembly patterns, supporting the view that seed microhabitats impose strong filtering on community assembly (Eyre *et al.*, 2019). In rice, distinct bacterial and fungal assemblages were detected across four compartments (grain, outer grain, husk, and outer husk), and “core” membership (which refers to taxa consistently detected across seed lots within the defined compartment in that study) differs by compartment (Eyre *et al.*, 2019). Seed compartment structure may also provide insight into transmission routes. For example, in pedunculate oak (*Quercus robur*), embryo-associated communities most closely matched the seedling phyllosphere, whereas pericarp- and root-associated communities were less diverse and compositionally distinct. These findings suggest that embryo- and seed coat-associated microbes may contribute to seedling colonization through distinct transmission pathways (Abdelfattah *et al.*, 2021). Evidence for microbial inheritance is not restricted to bacteria. Fungi can also contribute to vertically transmitted components of the early plant microbiome, highlighting the importance of tracking both bacterial and fungal communities in studies of microbial inheritance (Vujanovic *et al.*, 2019; Johnston-Monje *et al.*, 2021).

New evidence suggests that seed microbiota can contribute to transgenerational stress legacies (that is, parental environment–dependent microbial states that are associated with offspring seeds and correlate with offspring performance under stress). In wheat, Azarbad *et al*. explicitly separated seed epiphytes from seed endophytes and combined multi-year rainfall manipulation field experiments to test whether seed-associated communities transmit drought legacies across generations (Azarbad *et al.*, 2026). In their field experiment, reduced rainfall initially decreased yield in the drought-sensitive wheat cultivar (AC Nass) while selecting for distinct seed bacterial endophyte communities. In subsequent generations, offspring whose seed microbiota retained compositional similarity to these drought-adapted communities showed enhanced yield stability under subsequent water stress (Azarbad *et al.*, 2026). However, identifying the microbial cause of this yield stability is difficult because the maternal environment can induce heritable host changes, including epigenetic modifications such as DNA methylation and histone marks (Labra *et al.*, 2002; Zhong *et al.*, 2009; Wang *et al.*, 2011; Raza *et al.*, 2025). These host-internal changes occur during seed development and can influence offspring phenotypes independently of the microbiome. Therefore, we suggest that future studies on vertical transmission consider: (i) use compartment-targeted manipulations to separate epiphyte-driven from endophyte-driven effects (for example, surface sterilization followed by defined re-inoculation, and embryo-focused delivery where feasible), and (ii) pairing microbiome manipulation with the characterization of heritable host programming (for example, methylome profiling) to test whether microbial effects are independent or covarying. These approaches will help separate the effects of transmitted bacteria from host-internal epigenetic programming.

### 2.2 Horizontal transmission

Horizontal acquisition (i.e., environmentally driven acquisition of microbial taxa) is expected to contribute substantially to seed microbial diversity because during both the plant development stage and seed dispersal, seeds encounter multiple external reservoirs (e.g., soil, air/dust, rainfall, insects; Fig. 1) (War et al., 2023; Romão *et al.*, 2025). For non-crop plants, seed dispersal processes can further influence microbial exposure by moving seeds among microsites. For example, animal-mediated dispersal and post-dispersal handling or predation can generate non-random “deposition networks” that redistribute seeds across habitats (McConkey *et al.,* 2026). Wind-driven dispersal can likewise alter seed trapping and retention across microsites, which should alter exposure to local dust and soil-associated sources even before germination (Ray *et al.,* 2026).

**Fig. 2:**
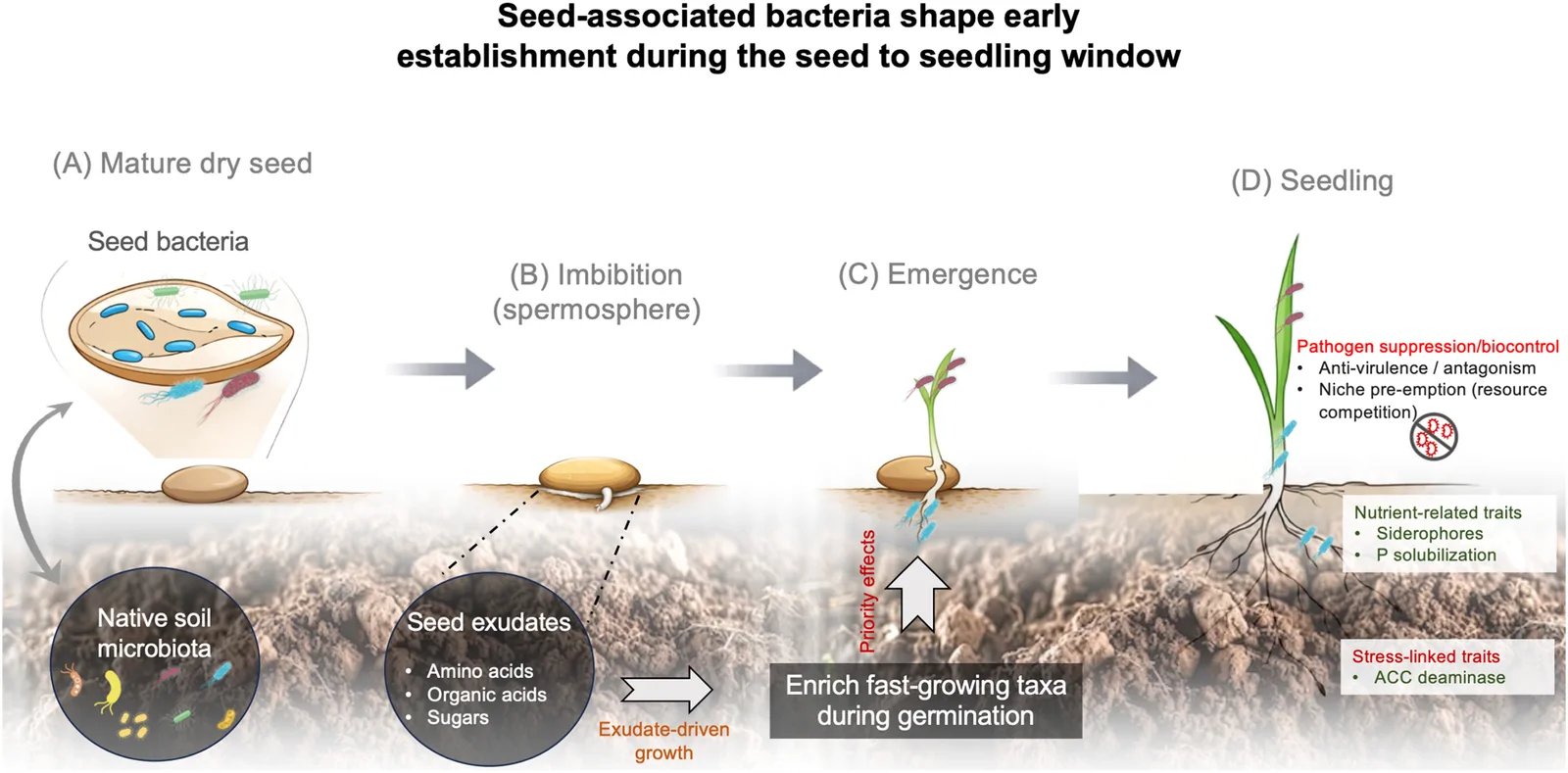
Seed-associated bacteria influence germination and seedling establishment during the seed-to-seedling transition. Dry seeds carry seed-surface and internal bacteria that become active during imbibition (A). As soon as seeds enter the soil environment, they are exposed to native soil microbiota and need to compete with diverse soil bacteria and fungi communities. During imbibition, seed exudates (including sugars, amino acids, and organic acids) generate a short-lived spermosphere (B) that can enrich fast-growing taxa and create priority effects during germination and emergence (C). During early seedling establishment (D), seed-associated bacteria can contribute to (i) pathogen suppression (for example, anti-virulence/antagonism and niche pre-emption), (ii) nutrient acquisition traits (for example, siderophores and nutrient mobilization), and (iii) stress-linked traits (for example, ACC deaminase–linked modulation of ethylene signalling). Functions shown represent experimentally supported examples highlighted in Sections 3.1–3.3.

Direct evidence for horizontal acquisition during seed development comes from rice. Using qPCR, 16S profiling, and microscopy, Chen *et al*. showed that bacterial acquisition peaked around panicle heading and flowering. Many acquired bacteria originated from the external environment and localized between the caryopsis and glumes. Their detection in seedlings grown under sterile conditions supports the vertical persistence of horizontally acquired bacteria from seed to seedling (Chen *et al.*, 2024). Once seeds contact non-sterile soil, seed and soil communities coalesce, meaning that they mix and reassemble (Fig. 2). This process is often asymmetric, with soil taxa dominating and few seed-borne taxa persisting. In *Brassica napus*, only a small fraction of seed-borne taxa colonized seedlings in soil (Rochefort *et al.*, 2021), suggesting why seed-stage community assembly patterns do not necessarily persist after sowing. After sowing, germination changes the surrounding seed environment. Seed exudates impose nutrient-driven selection as seeds move from a dry, low-activity state to an actively metabolizing state, which may in turn impact microbial communities (Han *et al.*, 2025). Imbibition (initial water uptake by dry seed) and early reserve mobilization (the start of using stored carbon and nitrogen to encourage emergence) create a transient nutrient pulse that can favour fast-growing, resource-responsive taxa (Fig. 2). Metagenomic predictions and isolate assays showed that bacteria enriched during seedling emergence had greater growth potential on seed exudate substrates than depleted taxa (Torres-Cortés *et al.*, 2018). Accordingly, fast-growing bacterial genera (e.g., *Pantoea, Enterobacter, Erwinia, Rahnella*) were enriched, whereas seed-associated lineages with lower growth potential (e.g., *Staphylococcus, Carnobacter*, and *Sanguibacter*) were depleted (Torres-Cortés *et al.*, 2018). In soybeans, Gerna *et al*. separated imbibition from germinative metabolism by using exogenous ABA to suppress germination while permitting hydration, although direct ABA effects on bacteria could not be excluded. Imbibition redistributed seed-coat-derived bacteria across seed compartments, whereas germinative metabolism increased richness and favoured copiotrophic taxa (Gerna *et al.*, 2024). *Pantoea agglomerans* also grew faster than *Rhodococcus fascians* on metabolite-informed media, supporting growth-rate differences as one contributor to community reassembly (Gerna *et al.*, 2024).

Together, these studies support a mixed transmission model (as shown in Fig. 1): a small subset of seed-associated lineages can be detected in both parent and offspring seeds (Section 2.1), whereas much of seed-associated diversity is acquired from environmental sources and then strongly re-filtered during germination and, especially, after soil contact. A major limitation is that many studies sample only one endpoint (for example, seeds or seedlings) rather than quantifying the full sequence of acquisition → maturation (dry seeds)/storage → germination → soil exposure. To address this, we propose that future research reports outcomes across four successional stages: (i) acquisition into developing seeds (including entry timing and localization during reproductive development), (ii) persistence through maturation and storage (including changes in absolute microbial load and viability), (iii) proliferation during germination, defined explicitly as an increase in absolute abundance of specific taxa from dry seed to imbibed/germinated states (not only changes in relative abundance), and (iv) establishment after soil exposure, quantified as the fraction of seed-borne taxa that remain detectable in seedlings after seed–soil mixing under realistic conditions (Rochefort *et al.*, 2021; Gerna *et al.*, 2024). Finally, direct experimental evidence is still needed to determine whether distinct seed microhabitats, such as the grain interior and husk or outer layers, preferentially colonize specific seedling compartments under axenic and soil conditions. Although rice seeds show strong compartment-specific community structure, the routes by which these compartments contribute to seedling colonization are still rarely tested with compartment-targeted tracking or manipulation (Eyre *et al.*, 2019).

## 3 Functional roles of seed-associated bacteria during the seed-to-seedling transition

In this section, we focus on three experimentally tractable outcomes commonly reported in seed microbiome studies: (i) effects on germination timing and early growth, (ii) modulation of nutrient- and stress-linked traits during establishment, and (iii) early protection against pathogens.

### 3.1 Enhancement of germination and early growth

A fraction of the seed microbiota remains viable but metabolically inactive or exhibits very low metabolic activity in dry seeds and becomes active during germination, yet the cues that trigger this transition remain poorly understood and require direct mechanistic tests (Gerna *et al.*, 2025). During imbibition and early reserve mobilization, seeds shift from a dry, low-activity habitat to a nutrient-rich microenvironment enriched with seed-derived exudates (Schiltz et al., 2015; Han *et al.*, 2025) that can restructure seed-associated microbiota by favoring fast-growing taxa capable of utilizing these released substrates (Torres-Cortés *et al.*, 2018; Gerna *et al.*, 2024). In rice, hybrid seeds (TFHZ, ZYH7, HZ261) carried distinct endophytic communities compared with their parental lines, and inoculation assays using endophyte isolates from hybrids resulted in greater improvements in germination-related traits than inoculation with isolates derived from the parents (Liu *et al.*, 2024). Perturbation–rescue designs strengthen causal inference by showing that removing resident seed microbes impairs early performance and that reintroducing defined seed-derived strains can restore part of that plant phenotype. In maize (var. SHS 5050), sodium hypochlorite disinfection (1.25% NaClO, 30 min) reduced the resident seed bacterial community and delayed germination and seedling growth in a gnotobiotic system. However, re-inoculation with a seed-derived synthetic community partially restored seedling establishment, supporting a direct contribution of seed-borne bacteria to early plant performance (Figueiredo dos Santos *et al.*, 2021). In a multi-species forage survey, Dai *et al*. isolated *Bacillus subtilis* (Es-1) and *Pantoea agglomerans* (Ed-3) and tested effects on germination rate and seedling fresh weight across hosts, illustrating that seed-derived isolates can shift early growth metrics but that effects vary across plant species (Dai *et al.*, 2020). Finally, seed endophytes may contribute to early growth beyond crop models. In the Pb–Zn hyperaccumulator *Noccaea caerulescens*, seed endophyte profiling across polluted sites suggested broadly conserved community structure, and inoculation with a cultured seed isolate (*Sphingomonas vulcanisoli*) increased root length in a model plant assay, providing a tractable isolate-level entry point for testing seed-borne functions under metal-stress contexts (Langill *et al.*, 2023). Together, these studies support germination as an experimentally tractable window (Fig. 2) in which (i) seed physiology alters the resource landscape, (ii) early seeds or soil-derived bacteria can gain priority during colonization, and (iii) microbial activity can impact plant phenotypes (e.g., germination timing, early biomass).

### 3.2 Nutrient mobilization and stress mitigation

Seed-associated bacteria can influence nutrient- and stress-relevant traits during early establishment of plants via strain-specific functions that modulate micronutrient availability and plant stress signalling. A stress-context example comes from *Vicia sativa* (cv. Lanjian No. 3), where Xu *et al*. profiled seed-derived communities after cold, salt, or PEG-simulated drought stress and separated taxa into stress-generalists (e.g., *Methylobacterium, Pantoea*, and *Sphingomonas*) versus stress-specialists (e.g., *Stenotrophomonas, Geobacter*, and *Leptotrichia*). They then constructed inocula from cultured representatives and showed that “generalist” inocula improved seedling biomass across multiple stress conditions, whereas “specialist” inocula performed best when matched to the corresponding stress (Xu *et al.*, 2026*a*). These findings support the idea that strain selection should be tailored to the target stress rather than assuming the existence of a single “universal” seed biostimulant. Beyond effects on plant growth, seed-endophyte inoculation studies have also reported changes in ion homeostasis, a physiological trait closely associated with stress tolerance. In barley (*Hordeum vulgare*), Abideen *et al*. inoculated plants with two seed-derived endophytes (identified as *Pseudomonas* sp. and *Pantoea* sp.) and quantified mineral ion accumulation under drought conditions. Inoculated plants exhibited higher shoot and root K⁺ concentrations and higher K⁺/Na⁺ ratios than uninoculated controls (Abideen *et al.*, 2022).

Seed microbiota from extreme habitats also suggests strong environmental filtering during seed colonization. In the holoparasitic plant *Cistanche armena* (saline, arid habitat), Petrosyan *et al*. reported that culturable seed endophytes were dominated by spore-forming, halotolerant/alkaliphilic *Bacillus* spp., and they identified *Pantoea* and *Stenotrophomonas* isolates with plant growth–promoting traits, including auxin production, ACC deaminase activity, and organic-acid production (Petrosyan *et al.*, 2022). In tomato (*Solanum lycopersicum*), Dargiri and Samsampour pretreated surface-sterilized seeds by immersing them in fungal spores and/or bacterial suspensions (supplemented with 1% carboxymethyl cellulose to enhance contact), then grew seedlings in autoclaved peat/perlite and quantified traits at the four-leaf stage (45 days). They reported that several fungal–bacterial combinations increased growth metrics (fresh/dry biomass, height) and shifted multiple plant physiological/biochemical traits, including chlorophyll/carotenoids, photosystem II efficiency parameters, phenolics, antioxidant activity, proline, and carbohydrates (Dargiri and Samsampour, 2025). This supports the feasibility of multi-kingdom consortia as seed-delivered stress modulators (Azarbad and Junker, 2024; Dargiri and Samsampour, 2025). Collectively, the most seed-relevant evidence supports a strain-centred model in which specific culturable microbes can shift nutrient- and stress-linked traits during the seedling period. However, establishing field relevance will require quantifying absolute colonization dynamics from seeds to specific seedling compartments and re-evaluating candidate strains in non-sterile soil, where competition and dilution are expected to be greatest.

### 3.3 Pathogen suppression and biocontrol

Seed-associated microbes can reduce pathogen pressure during seedling emergence because they are already present at the seed–seedling transition, when pathogen infection and competitive sorting are strongest (Díaz Herrera *et al.*, 2016; Ajayi *et al.*, 2024; Hu *et al.*, 2025). Here, biocontrol refers to protection driven by microbes that either inhibit pathogens directly (for example, by antifungal activity) or indirectly (for example, by occupying resources or space that pathogens need). In barley, Ajayi *et al*. isolated bacterial seed endophytes and showed in vitro inhibition of *Fusarium graminearum*, supporting the idea that seeds can carry antagonistic strains (Ajayi *et al.*, 2024). In radish (*Raphanus sativus*), the core seed microbiota maintains taxonomic stability during the introduction of the pathogen *Xanthomonas campestris* (Xcc8004) because the pathogen utilizes carbon sources that do not overlap with those of the primary residents. By contrast, native *Stenotrophomonas rhizophila* strains showed greater resource overlap with the pathogen and were proposed as candidates that could limit pathogen establishment through niche pre-emption, effectively “getting there first” and occupying the resources the pathogen would need (Torres-Cortés *et al.*, 2019). While niche pre-emption depends on resource depletion, seed-borne protection also can function through the targeted disruption of pathogen signaling. In rice (*Oryza sativa*), the seed-transmitted endophyte *Sphingomonas melonis* confers resistance to the seedling blight pathogen *Burkholderia plantarii*. This protection was mediated by the production of anthranilic acid, a metabolite that attenuates pathogen virulence by interfering with RpoS-dependent regulation (Matsumoto *et al.*, 2021).

Beyond targeted signaling interference, the seed microbiota provides broad-spectrum defense through diverse antagonistic lineages and community-level suppressiveness. In seedling assays, seed-derived *Bacillus cereus* and *B. toyonensis* directly inhibit *Fusarium solani* and *F. oxysporum*, reducing root-rot symptoms while enhancing germination and seedling biomass (Hu *et al.*, 2025). Similarly, the rice seed endosphere hosts antagonistic *Burkholderia* and *Pantoea* strains that suppress seedling blight fungi, providing a functional source of candidates for seed-applied biocontrol (Khanal *et al.*, 2024). In spinach (*Spinacia oleracea*), natural suppressiveness against *Globisporangium ultimum* damping-off was associated with enrichment of *Massilia* and basidiomycetous yeasts, such as *Vishniacozyma* and *Filobasidium* (Diakaki *et al.*, 2025). In potato (*Solanum tuberosum*), the taxonomic composition of the seed tuber microbiome can enable the high-throughput modeling of crop vigor when integrated with aerial multispectral imaging (Song *et al.*, 2025).

Collectively, these findings position the seed microbiota as a biological component where specific seed microbes and/or community signatures may enhance plant traits. However, a primary gap in current research is the disconnect between laboratory-based antagonism and the actual ecological persistence of protective taxa during host establishment. Most studies rely on in vitro assays or simplified seedling models that fail to track whether protective strains successfully colonize and persist in the plant over time. To bridge this gap, studies should consider providing spatial resolution of antagonistic mechanisms, specifically distinguishing between competition on the outer seed coat, colonization and occupancy of the internal endosphere, and suppression within the surrounding soil-seed interface. This level of detail is required to determine if seed microbes can withstand the metabolic and taxonomic pressures of soil-derived community integration.

## 4 Host genotype, domestication, and environmental effects

This section details the host genetics that control microbial recruitment and analyzes how these inherited pathways were restructured during crop domestication. We further evaluate maternal environmental stress and post-harvest storage as selective barriers that determine which seed-borne bacteria remain viable and can colonize the emerging seedling. Together, these cumulative layers of selection define the taxonomic composition and functional potential of the microbial lineages available for vertical transmission and early seedling establishment.

### 4.1 Influence of host genotype and breeding

Host genotype can shape community assembly of the seed-associated microbiota (Wassermann *et al.*, 2022; Ajayi *et al.*, 2024), but the magnitude of this effect depends on whether the study targets the seed endophytes versus the seed epiphytes (Azarbad *et al.*, 2022, 2026). In winter oilseed rape (*B. napus*), the seed bacterial community structure differed among cultivars, and cultivar-specific responses were also observed in surface-sterilized assays using a defined strain. For example, *Pseudomonas brassicacearum* (CKB26) showed cultivar-dependent colonization of seeds, and seedling growth responses differed among cultivars (Rybakova *et al.*, 2017). Beyond differences between plant varieties, specific breeding history, such as hybridization, also determines the composition of the internal bacterial reservoir. In rice (*Oryza sativa*), hybrid seeds contain a different set of endophyte bacteria than their parent plants. These microbial changes result in direct benefits for the next generation, where bacteria isolated from hybrid seeds were more effective at promoting germination than those from the parental lines (Liu *et al.*, 2024).

The transition from wild emmer (*Triticum dicoccoides*) to domesticated bread wheat (*T. aestivum*) resulted in a reduction in seed-borne bacteria richness and taxonomic diversity (defined as the frequency of specific taxa across individual seeds). While wild emmer maintains a predictable microbial profile, domesticated varieties exhibit high inter-seed stochasticity. Gnotobiotic assays further confirmed that modern breeding has produced initial seed-associated microbiota that is less diverse, providing a less reliable colonizing source for modern wheat progeny than that of its wild ancestors (Özkurt *et al.*, 2020). These empirical results validate the framework of microbiome gene (M gene) breeding, identifying host genetics as a primary target for improving crop health through microbial selection (Cernava, 2024). Within this framework, the host’s capacity to recruit beneficial microbes is treated as a selectable trait, distinct from traditional resistance or susceptibility genes. By targeting the specific host genes that regulate the vertical transmission of the seed microbiome, breeders can intentionally select for cultivars with more stable and functional biological defenses (Cernava, 2024).

### 4.2 Domestication and microbiome evolution

Domestication of cereals, specifically wheat, barley, and rye, has been shown to drive a significant increase in seed-borne bacterial diversity while concurrently reducing the complexity and connectivity of seed microbial co-occurrence networks (Abdullaeva *et al.*, 2021). This taxonomic diversification in cultivated crops was characterized by the enrichment of generalist, human-associated taxa such as *Cutibacterium*, contrasting with the more structured and stable microbial assemblies found in wild ancestors (Abdullaeva *et al.*, 2021). These structural changes extended to the seedling stage, where breeding history determined the competitive balance between inherited and environmental microbes. In wheat, wild ancestors exhibited higher colonization efficiency for vertically transmitted taxa, whereas modern cultivars relied more extensively on recruitment from soil microbial reservoir to populate the early rhizosphere and endosphere (Abdullaeva *et al.*, 2022). A similar domestication-linked restructuring was observed in *Cannabis sativa*, where intensive breeding reduced the taxonomic diversity and increased the homogeneity of seed-borne bacterial communities (Lobato *et al.*, 2024). This shift was evidenced by a significant decrease in bacterial richness, with modern hybrids showing lower Shannon diversity than ancestral landraces (Lobato *et al.*, 2024). These shifts were associated with the loss of specialized defensive endophytes that protected the emerging seedling from soil-borne pathogens. In maize (*Zea mays*), seeds from native landraces carried a significantly higher endophytic microbial abundance and greater taxonomic diversity than modern hybrid varieties (Gastélum *et al.*, 2022). Functional screening through pairwise interaction assays established that landrace-derived endophytes possessed enhanced competitive traits, specifically through high-frequency antagonism against soil bacteria that was largely absent in modern hybrid isolates. The most potent antagonistic strains were identified as *Burkholderia* spp., a genus detected exclusively in native landrace seeds (Gastélum *et al.*, 2022).

Experimental introduction of *Paraburkholderia phytofirmans* PsJN via floral spraying bypassed host barriers to colonize the seed embryos of both monocots (maize, wheat) and dicots (soybean, pepper) (Mitter *et al.*, 2017). In wheat (cv. Trappe), this internal colonization accelerated reproductive development, leading to significantly earlier spike onset in the first-generation offspring. However, PsJN was not detected in the seeds of the subsequent generation, confirming that cross-host colonization and immediate functional benefits did not translate into stable vertical transmission (Mitter *et al.*, 2017). These results indicate that effective microbiome engineering depends on the delivery of specific functional traits and the selection of host genotypes capable of ensuring the transgenerational persistence of these microbial strains or consortia. Successful integration requires the host to maintain a stable microbiome across successive life cycles, transitioning the engineered members from transient inoculants to heritable components of the plant holobiont.

### 4.3 Maternal environment and stress modulation

The maternal environment (the biotic and abiotic conditions experienced by the mother plant during flowering, seed filling, and maturation) can shift seed-associated communities by changing (i) the microbes that can access reproductive tissues and developing seeds and (ii) within-seed conditions that determine which taxa persist to maturity (Zhou *et al.*, 2020; Sulesky-Grieb *et al.*, 2024; Azarbad *et al.*, 2026). In rice, field-scale sampling across environments reported that seed microbiota covaried with climate-linked variables (including seed moisture and winter temperature) (Zhou *et al.*, 2020). Agronomic management can also function as a maternal environment because it changes plant exposure histories (soil biota, agrochemical regimes, and field microclimate) during plant development (Azarbad *et al.*, 2022; Khanal *et al.*, 2024). In rice, comparison of conventional versus organic production reported higher endophytic bacterial diversity in conventionally produced seeds but higher fungal diversity in organically produced seeds (Khanal *et al.*, 2024). Functional screens and inoculation assays suggest that maternal/stress context can also bias enrichment toward strain-level candidates with measurable drought-linked effects. In wheat, seed-associated isolates from drought-tolerant plants (including *Curtobacterium flaccumfaciens* and *Arthrobacter* spp.) improved seedling growth under drought in controlled experiments (Hone *et al.*, 2021). Many studies pool seed bacteria, obscuring whether stress effects originate on the seed surface or within tissues, and which fraction, as part of the maternal, actually transfers to seedlings. Compartment-specific quantification and targeted perturbations (surface depletion/re-inoculation versus internal delivery) are therefore needed to connect maternal environments to defined seed fractions and to test which fraction influences plant phenotypes. In wheat, Azarbad et al. separated seed epiphytes and endophytes and linked drought history to shifts in endophytic communities (Azarbad *et al.*, 2026).

### 4.4 Influence of seed storage conditions

Postharvest handling contributes to the restructuring of seed-associated bacterial communities because drying and storage impose time- and temperature-dependent filters on which taxa remain viable and detectable in stored seeds. In rice (*Oryza sativa* L.), Dutta *et al*. tracked seed endophytic communities during ripening and then stored harvested seeds from two cultivars (Shindongjin and Sukwang) at 4 °C versus 15 °C. They quantified culturable bacteria and profiled community composition over time, showing that culturability changed across ripening and storage, and that storage temperature was associated with differences in the seed bacterial community detected after storage (Dutta *et al.*, 2022). Importantly, when seedlings were grown under axenic conditions, the bacterial composition in shoots and roots differed depending on whether seeds had been stored at 4 °C or 15 °C (Dutta *et al.*, 2022). Additional evidence indicates that drying itself can be a strong perturbation rather than a neutral preprocessing step. In soybean (*Glycine max*), Chandel *et al*. applied a standard drying protocol (15 °C, 15% relative humidity, 1 month) prior to storage and observed that drying shifted the relative abundance of several dominant genera, including large changes in *Pseudomonas, Pantoea, Curtobacterium, Sphingomonas* and *Massilia* (Chandel *et al.*, 2021). Subsequent storage temperature then influenced how diversity was retained over time: across the temperatures tested (−20 °C, 4 °C, room temperature), −20 °C generally slowed diversity loss compared with warmer conditions, while some taxa showed temperature sensitivity (for example, *Massilia* declined under cold storage in that dataset) (Chandel *et al.*, 2021). Together, these studies justify reporting drying and storage parameters as important variables (temperature, humidity, duration), because they can measurably reshape which seed-borne bacteria are available to colonize the germinating seedling.

## 5 Bacterial isolates, culturability, and implications for seed microbiome engineering

Microbiome engineering refers to experimentally guided modification of a host-associated microbial community to change its assembly or function (Mueller and Sachs, 2015; Vorholt *et al.*, 2017; Ke *et al.*, 2021). In practice, this is often done by introducing defined strains as SynComs or consortia so that individual members can be added, removed, or substituted to test the relationship between microbial community composition and plant outcomes under controlled conditions (Vorholt *et al.*, 2017; Arnault *et al.*, 2024). A practical constraint for microbiome engineering is culturability, defined as the ability to recover plant-associated microbes as living isolates under defined laboratory conditions (Sarhan *et al.*, 2019; Mapelli *et al.*, 2023). This matters because strain-level functions (growth traits, metabolite production, stress tolerance) require isolates, not amplicon sequences alone (Riva *et al.*, 2022). Across plant endosphere studies, culture collections have been shown to recover only a small set of genera (*Bacillus* and *Pseudomonas*) across hosts and tissues, while many sequencing-detected lineages remain rarely isolated (Riva *et al.*, 2022). Seed-focused datasets illustrate how large this gap can be under standard cultivation methods (Cope-Selby *et al.*, 2017; Heitmann *et al.*, 2021; Chen *et al.*, 2022; Becker *et al.*, 2024). In highland barley seeds, culture-dependent isolation from surface-sterilized seeds yielded 86 isolates, the majority assigned to *Bacillus*, whereas 16S rRNA profiling of the same seed material was dominated by *Proteobacteria* and, at finer resolution, by *Enterobacteriaceae*. This mismatch was interpreted as being consistent with cultivation bias (Chen *et al.*, 2022).

Seed-system studies show that culturability of microbes can highly depend on the culturing strategy. Seed-context media can expand the fraction of the community that becomes culturable. In germinating soybean (*Glycine max*), Gerna *et al*. designed multiple solid media to mimic the seed endosphere and identified 246 isolates; they also reported that the embryonic axis harbored higher richness and more unique genera than cotyledons, highlighting the fact that “what you can culture” depends on which seed compartment you sample (Gerna *et al.*, 2022). They further showed that distinct seed lots yielded few overlapping taxa, consistent with strong lot-to-lot variability in the culturable endophytic pool (Gerna *et al.*, 2022). Isolation priorities can be made more “engineering-relevant” when they are guided by where and when bacteria accumulate in seeds and when they can be transferred to seedlings. For example, mapping seed microhabitats and identifying compartments associated with downstream seedling colonization provides a rational basis for selecting isolates that are more likely to matter at the seed to seedling transition, rather than assembling microbial consortia dominated by easy-to-grow taxa (conceptually aligned with the compartment-dependent patterns emphasized in seed studies such as Chen *et al.* 2024).

Seed inoculated with *Bacillus subtilis* has been tested across crop–stress settings (including maize under salinity and wheat under drought) with measurable improvements in early seedling traits in controlled assays, but outcomes vary with the specific strain, dose, and test system (Hu *et al.*, 2019; Song *et al.*, 2023). Likewise, seed/seedling applications of *Pseudomonas fluorescens* have been reported to enhance germination/seedling vigor and contribute to disease suppression in crop establishment contexts, again with performance contingent on formulation and strain properties (Lally *et al.*, 2017; Rivera-Conde *et al.*, 2018; Nilmat *et al.*, 2023). Seed microbiome engineering should not default to a narrow set of “easy to culture genera” because seed-relevant studies also include strain-level examples beyond *Bacillus* and *Pseudomonas*. For example, the wheat-associated *Variovorax* isolate (P1R9) improved germination and early seedling traits when applied alone or as part of consortia (Acuña *et al.*, 2024). Similarly, responses to seed inoculation with *Azospirillum brasilense* depend on both inoculant strain and seed traits such as seed size, illustrating that host traits can modulate bacterial efficacy at the earliest development stages (José *et al.*, 2024). Moving forward, these examples provide valuable evidence to quantify intra-species strain diversity and test whether persistence and function in the plant environment can be due to strain-specific rather than genus-level properties.

## 6 Synthetic community (SynCom) for seed microbiome engineering

**Fig. 3:**
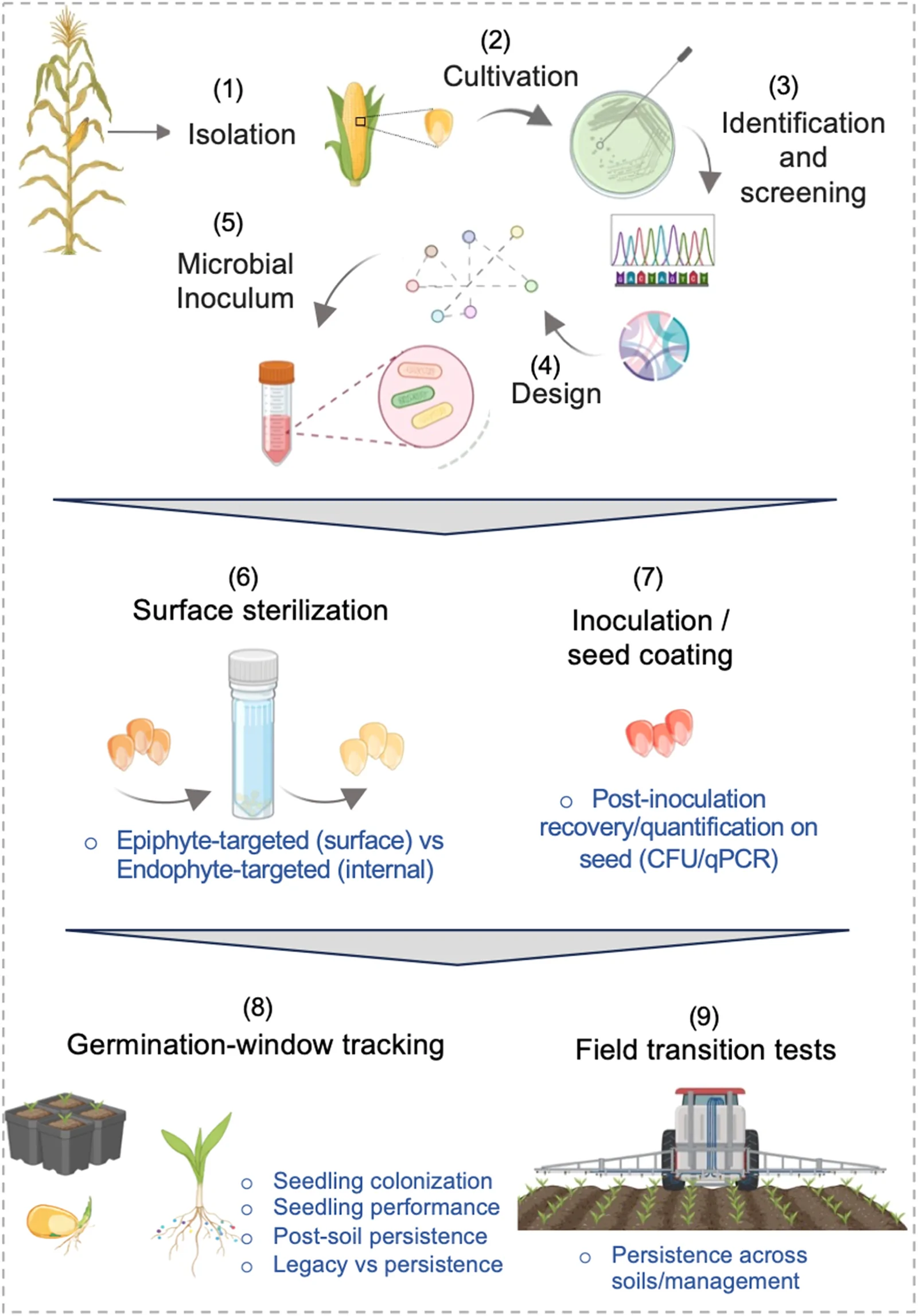
A seed-focused workflow for synthetic community (SynCom) engineering and evaluation. Seed-associated strains are isolated (1), cultivated (2), and identified and screened (3) before SynCom design (4) and preparation of a defined microbial inoculum (5). Seeds are then prepared in ways that distinguish surface-associated (epiphytic) from internal (endophytic) fractions (6), followed by seed inoculation/seed coating (7). Performance is first assessed during the germination window with strain/community tracking and the characterization of early seedling physiological and morphological traits (8), and then tested for robustness in field transition experiments, ideally across sites and soils (9).

Seed SynComs can help to test which seed-borne strains alter seedling microbial community assembly and plant traits, and at which stage those effects appear (Portal-Gonzalez *et al.*, 2025; Xu *et al.*, 2025). Under this section, we present a seed-focused workflow for SynCom (Fig. 3).

*Strain selection and SynCom design*. Seed-focused SynComs usually start by isolating and culturing seed bacteria (Section 5). To maximize functional potential, selection should draw from isolates recovered from both the seed surface and internal compartments, ensuring a complete representation of the seed-associated microbiota. Where possible, SynCom designs should include within-genus diversity (multiple strains from the same genus) to test whether colonization and plant responses are strain-specific rather than general to a taxonomic label. SynCom design then uses defined membership and richness to test how microbial community context influences the assembly of the seedling microbiota. In radish, SynCom experiments showed that seed-to-seedling transmission differed strongly among strains and shifted with community richness, and that seedling trait distributions (including abnormal seedlings) also depended on community context (Simonin *et al.*, 2023). These results may support using richness gradients not to claim that “more diversity is better”, but to tease apart which strains are robust colonizers, which are sensitive to community context, and which drive positive or negative early phenotypes under controlled conditions.

*Seed sterilization and coating.* Surface sterilization is widely used to reduce background variation and to enable strain tracking, but it also removes part of the resident epiphytic community and can change the starting conditions. Sterilization steps should therefore be reported and justified, and paired with appropriate controls (Azarbad and Junker, 2024). Because seed coating/inoculation sets the starting abundance of each strain, studies should report inoculum density and confirm post-inoculation recovery on seeds (for example, CFU per seed). This matters because starting abundance can strongly shape which strains establish after germination (Arnault *et al.*, 2024). A complementary design is to inoculate seeds without removing the resident microbiota, which tests whether a SynCom can act in the presence of the native seed community. In lucerne, Dissanayakalage *et al*. inoculated non-sterilized seeds and observed treatment-associated shifts in seedling traits and bacterial community profiles (Dissanayakalage *et al.*, 2025). However, without strain tracking, the data cannot distinguish between the persistence of the inoculated strains and indirect effects mediated by the resident community.

*Germination-window tracking and early outcomes*. Under controlled laboratory conditions, seed-applied SynComs can strongly shape seedling microbial communities, which is consistent with priority effects (early colonists gain an advantage because they occupy emerging niches first) (Arnault *et al.*, 2024). A mechanistic explanation is that germination releases a short-lived pulse of seed exudates that increases local resource availability around the seed and favors fast-growing strains (Torres-Cortés *et al.*, 2018). This is why seed SynCom studies should quantify both colonization dynamics and plant outcomes across the germination window, rather than reporting a single endpoint. Beyond external inoculation, “vertical-entry” approaches aim to establish strains inside seed tissues. In wheat, labelled endophyte experiments support the feasibility of tracking an introduced lineage through the plant life cycle and detecting it again in offspring seeds, indicating that seed-mediated persistence is possible when internal establishment is achieved (Sanz-Puente *et al.*, 2025).

*Field transition, durability, and life-cycle effects.* The main barrier to translation from controlled assays to soil-grown plants (in particular under field conditions) is that effects observed during germination often weaken after soil exposure, when seed- and soil-derived communities mix and reassemble, and soil taxa become dominant (Rochefort *et al.*, 2021). Although full plant life-cycle and multigenerational tests are less studied, available seed-inoculation work suggests that SynCom members can become rare (or even become not detectable) later in plant development while still leaving legacy effects on community assembly and/or plant performance, including signals detectable in the next seed generation (Arnault *et al.*, 2025). Therefore, it is highly important to treat persistence (continued detectability of introduced strains) and legacy effects (downstream shifts that remain after introduced strains become rare or undetectable) as separate outcomes because they imply different mechanisms and different design targets (Arnault *et al.*, 2025). Garrido-Sanz and Keel designed a reproducible wheat rhizosphere community in microcosms and reported that a “heritable seed-borne rhizosphere microbiome” can surpass native soil microbes as the dominant rhizosphere source in their system. They link this to (i) niche partitioning (meaning taxa specialize on different substrates, reducing direct competition) and (ii) facilitation/cross-feeding (meaning one taxon enables another’s growth). This kind of mechanism is the level of evidence SynCom studies should aim for when claiming predictable SynCom design (Garrido-Sanz and Keel, 2025).

## 7 Outlook: seed microbiome engineering for sustainable agriculture

A practical way to compare seed-SynCom studies is to report outcomes using the same step-by-step workflow as discussed in Section 6. At minimum, studies should separate: (i) initial establishment (detectable on or within the seed), (ii) proliferation during germination (an increase in absolute abundance of a strain, not only a change in relative abundance), (iii) seedling organ colonization (root and shoot/leaf), and (iv) persistence after soil exposure (continued detectability after seed–soil mixing). Beyond the promotion of seedling emergence, yield buffering and crop quality remain underdeveloped for seed microbiome engineering. Soil microbiome features can predict wheat baking-quality traits at harvest, supporting microbiome-informed quality forecasting but not establishing a seed-microbiome mechanism (Asad *et al.*, 2023). This raises a seed-focused question: can engineered seed microbial communities shift end-product quantity and quality (for example, nutritional composition or processing traits), and if so, do the responsible microbes need to persist beyond the germination window, or can early-life effects be sufficient? Addressing this will require defined seed microbiome manipulation coupled with microbial tracking across plant development and standardized quantity and quality phenotyping at harvest.

## 8 Conclusion

Seed-associated bacteria support plant establishment and resilience by facilitating nutrient mobilization and suppressing environmental pathogens. These microbial communities are initiated during the flowering phase, consolidated throughout seed development, and reactivated at germination to early seedling microbial community assembly and the initial establishment of the plant holobiont. The last decade has revealed that seed microbiota is heritable, dynamic, and manipulable, shaped by host genetics, maternal environment, and domestication. While selective breeding and modern seed sanitation have unintentionally narrowed microbial diversity, the emergence of microbiome-assisted breeding and SynCom-based inoculation provides new opportunities to restore and optimize these hidden allies. Looking ahead, the future of sustainable crop production may depend as much on microbial inheritance as on plant genetics. Ultimately, understanding and engineering the seed microbiome can transform the seed from a germplasm unit into a living biological vector that carries both host genetic and microbial legacies, essential for plant fitness and sustainable agriculture.

## CRediT authorship contribution statement

**Hamed Azarbad:** Writing – original draft, Writing – review & editing, Visualization, Validation, Resources, Project administration, Methodology, Investigation, Formal analysis, Conceptualization. **Mehrdad Alizadeh:** Writing – original draft, Writing – review & editing, Visualization, Validation, Resources, Project administration, Methodology, Investigation, Formal analysis, Conceptualization.

## Declaration of competing interest

The authors declare the following financial interests/personal relationships which may be considered as potential competing interests:
